# A Remote Intervention to Prevent or Delay Cognitive Impairment in Older Adults: Design, Recruitment, and Baseline Characteristics of the Virtual Cognitive Health (VC Health) Study

**DOI:** 10.2196/11368

**Published:** 2018-08-13

**Authors:** Nicholas Bott, Shefali Kumar, Caitlyn Krebs, Jordan M Glenn, Erica N Madero, Jessie L Juusola

**Affiliations:** ^1^ Department of Medicine School of Medicine Stanford University Stanford, CA United States; ^2^ Neurotrack Technologies, Inc Redwood City, CA United States; ^3^ Evidation Health San Mateo, CA United States

**Keywords:** cognitive impairment, dementia, Alzheimer disease, lifestyle intervention, digital health, health coaching, cognitive dysfunction, risk reduction behavior

## Abstract

**Background:**

A growing body of evidence supports the use of lifestyle interventions for preventing or delaying the onset of Alzheimer disease and other forms of dementia in at-risk individuals. The development of internet-delivered programs would increase the scalability and reach of these interventions, but requires validation to ensure similar effectiveness to brick-and-mortar options.

**Objective:**

We describe the study design, recruitment process, and baseline participant characteristics of the sample in the Virtual Cognitive Health (VC Health) study. Future analyses will assess the impact of the remotely delivered lifestyle intervention on (1) cognitive function, (2) depression and anxiety, and (3) various lifestyle behaviors, including diet, exercise, and sleep, in a cohort of older adults with subjective memory decline. Additional analyses will explore feasibility outcomes, as well as the participants’ engagement patterns with the program.

**Methods:**

Older adults (aged 60-75 years) with subjective memory decline as measured by the Subjective Cognitive Decline 9-item (SCD-9) questionnaire, and who reported feeling worried about their memory decline, were eligible to participate in this single-arm pre-post study. All participants enrolled in the yearlong digital intervention, which consists of health coach-guided lifestyle change for improving diet, exercise, sleep, stress, and cognition. All components of this study were conducted remotely, including the collection of data and the administration of the intervention. We assessed participants at baseline, 12 weeks, 24 weeks, and 52 weeks with online surveys and the Repeatable Battery for the Assessment of Neuropsychological Status (RBANS) test. We will conduct intention-to-treat analysis on all outcomes.

**Results:**

A total of 85 participants enrolled in the intervention and 82 are included in the study sample (3 participants withdrew). The study cohort of 82 participants comprises 61 (74%) female, 72 (88%) white, and 64 (78%) overweight or obese participants, and 55 (67%) have at least a college degree. The average baseline RBANS score was 95.9 (SD 11.1), which is within age-adjusted norms. The average SCD-9 score was 6.0 (SD 2.0), indicating minor subjective cognitive impairment at the beginning of the study. The average baseline Generalized Anxiety Disorder 7-item scale score was 6.2 (SD 4.5), and the average Patient Health Questionnaire 9-item score was 8.5 (SD 4.9), indicating mild levels of anxiety and depression at baseline.

**Conclusions:**

Internet-delivered lifestyle interventions are a scalable solution for the prevention or delay of Alzheimer disease. The results of this study will provide the first evidence for the effectiveness of a fully remote intervention and lay the groundwork for future investigations.

**Trial Registration:**

ClinicalTrials.gov NCT02969460; http://clinicaltrials.gov/ct2/show/NCT02969460 (Archived by WebCite at http://www.webcitation.org/71LkYAkSh)

**Registered Report Identifier:**

RR1-10.2196/11368

## Introduction

### Lifestyle Interventions for Cognitive Decline

Cognitive impairment is a growing public health epidemic worldwide, and is one of the most prevalent chronic medical conditions in older adults [1]. In 2010, the direct and indirect costs of care associated with dementia totaled US $600 billion globally, amounting to roughly 1% of the world’s gross domestic product [2]. The global costs associated with Alzheimer disease, the most common form of dementia, are projected to increase by about 400% from US $186 billion in 2018 to US $750 billion in 2050 [1]. The challenge posed by dementia is amplified by the decades-long failure to develop effective pharmacologic agents for the disease. The success rate of Alzheimer disease drugs is only 0.4%, compared with 19% for oncology compounds [3], leading some major pharmaceutical companies to abandon research efforts in the face of continued failures. The drugs approved for Alzheimer disease treat only the symptoms rather than the underlying causes of the disease, and do not prevent or delay the progression of neurodegeneration involved in Alzheimer disease and other forms of dementia [4].

Conversely, nonpharmacologic lifestyle-based interventions are gaining traction as an effective way to prevent or delay disease progression. Epidemiologic studies estimate that modifiable risk factors, such as diabetes, hypertension, obesity, smoking, depression, physical inactivity, and low educational attainment, account for as many as 30% of dementia cases [5-7]. In the absence of a cure, delay of Alzheimer disease or dementia onset by as little as 1 year is associated with enormous cost savings, with an estimated potential savings of US $219 billion by 2050 in the United States alone [8]. Additionally, a 5-year postponement could almost halve the projected Alzheimer disease prevalence by 2050 [9-11]. Due to the proven ability to decrease some of the modifiable risk factors implicated in Alzheimer disease [12,13], lifestyle-based interventions hold the potential to greatly reduce the burden of dementia as populations continue to age worldwide.

The lifestyle intervention for cognitive decline used in the multidomain Finnish Geriatric Intervention Study to Prevent Cognitive Impairment and Disability (FINGER) [14] has been at the forefront of these Alzheimer disease-related behavioral modification efforts and served as the primary inspiration for the program used in this study. The landmark FINGER study is an ongoing randomized controlled trial (RCT) demonstrating the efficacy of a multidomain intervention as a preventive measure in older adults at risk for cognitive decline and dementia. The lifestyle intervention primarily focuses on exercise, diet, cognitive training, and management of vascular risk factors. The 2-year results [15] clearly showed that (1) individuals can be motivated to make long-term changes in their lifestyle to preserve cognitive function, and (2) a multidomain lifestyle intervention can improve composite cognitive performance at a 2-year follow-up.

The FINGER study, which used a clinic-based lifestyle intervention, was the first RCT to provide proof-of-concept that attending to lifestyle and vascular factors can protect against cognitive decline [15]. The success of the FINGER study has spawned numerous in-clinic replication studies around the globe, including the Singapore Intervention Study to Prevent Cognitive Impairment and Disability (SINGER) [16], Multimodal Intervention to Delay Dementia and Disability in Rural China (MIND-CHINA) [17], and United States Study to Protect Brain Health Through Lifestyle Intervention to Reduce Risk (US POINTER) [18]. However, face-to-face lifestyle change programs like the ones used in these studies are constrained by geographical and other logistical challenges, therefore warranting the exploration of internet-based programs that are better suited for widespread adoption in a real-world setting. The Maintain Your Brain study is a large-scale clinical trial in Australia aiming to demonstrate the efficacy of a fully digital, multidomain intervention at preventing cognitive decline [19]; however, results will not be available for some years. The Virtual Cognitive Health (VC Health) study described here is the first trial, to our knowledge, that explores the effectiveness of a commercially available digital lifestyle intervention aimed at preventing or delaying cognitive decline in at-risk older adults.

### Objective

The primary objective of the VC Health study is to investigate the feasibility and effectiveness of a remotely delivered multidomain intervention for the prevention or delay of cognitive impairment in older adults at increased risk of cognitive decline. Secondary analyses will assess the effectiveness of the program at ameliorating symptoms of depression and anxiety, which are both risk factors for Alzheimer disease [20,21]. Supplemental analyses will examine patterns of user engagement with the program and changes in various lifestyle behaviors. The yearlong intervention consists of 6 months of active multidomain lifestyle change and 6 months of habit reinforcement during the maintenance phase. The main components of the intervention are coach-directed exercise, nutritional guidance, cognitive training, and social engagement. We hypothesize that this digital intervention modeled after the pivotal FINGER study [15] will result in (1) significant improvements in composite cognitive performance and (2) positive changes in depression and anxiety levels. Here we report the study design and analysis plan, as well as baseline characteristics of the study population for the VC Health study.

## Methods

### Study Design

The VC Health study is a 52-week-long, prospective intention-to-treat, single-arm, pre-post, virtual nationwide clinical trial to evaluate the impact of the VC Health program on cognitive function and mental health in older adults in the United States.

While conventional clinical trials rely on in-person interactions for recruitment, screening, enrollment, data collection, and data monitoring, virtual clinical trials are enabled by advances in technology and digital health, allowing for fully remote participation in clinical trials [22]. For the VC Health study, we used an online study platform (Achievement Studies; Evidation Health Inc.; San Mateo, CA, USA) to screen, obtain consent from, and enroll participants into the study, as well as to collect and monitor study data and guide participants through the trial. The study was approved by the Solutions Institutional Review Board (Little Rock, AR, USA) and is registered with clinicaltrials.gov (NCT02969460). The study protocol was designed and written by investigators at Evidation Health, with input and review from the VC Health intervention team.

### Participant Selection and Recruitment

We recruited study participants through various digital platforms, including online patient communities, social media, and targeted advertisements across all 50 US states. Potential participants learned about the trial through a Web portal explaining the study details. Those who were interested in participating then completed an online screener that assessed their eligibility for the study.

Eligible participants were aged 60 to 75 years; endorsed subjective cognitive decline with worry as assessed by the validated Subjective Cognitive Decline 9-item (SCD-9) questionnaire [23] and the 1-item subjective cognitive decline with worry item (“Do you feel like your memory is becoming worse?” Possible responses were “No,” “Yes, but this does not worry me,” or “Yes, this worries me”) [24], which have been shown to have early predictive value for progression to mild cognitive impairment and Alzheimer disease [24,25]; had reliable access to a phone, text messaging, and the internet; and were interested in using a coaching program for cognitive health (Textbox 1). Study candidates were considered ineligible if they had a history of mental illness, substance abuse, learning disability, neurologic conditions, or dementia; had ophthalmologic or visual problems that would interfere with computer use; were already using a cognitive-training coaching program; or were currently pregnant.

### Enrollment and Study Procedures

Study candidates who met the eligibility criteria and who were interested in participating provided electronic informed consent on the online study platform, and then completed an online baseline assessment, which consisted of questions about demographic characteristics, lifestyle and overall health behaviors, Patient Health Questionnaire 9-item (PHQ-9), Generalized Anxiety Disorder 7-item (GAD-7) scale, and self-reported sleep, diet, and activity levels. Next, we asked study candidates to schedule a baseline Repeatable Battery for the Assessment of Neuropsychological Status (RBANS) test. The RBANS was remotely administered via video-teleconference by a licensed psychologist (Echelon Group; Woodstock, GA, USA). Once participants completed the RBANS test, we asked them to complete their first VC Health coaching session. This session lasted approximately 60 minutes and was conducted over the phone. We considered study candidates to be enrolled once they completed their first coaching session.

Once enrolled, participants completed online assessments and RBANS tests at 12 weeks, 24 weeks, and 52 weeks. Since there are four alternative forms of the RBANS test, designed to reduce practice effects during repeated testing over time, we gave participants in this study a different form for each of the 4 testing periods [26]. Participants were able to engage with the VC Health program throughout the 12-month study period.

**Inclusion and exclusion criteria for the VC Health study.**

Inclusion criteria

- Aged ≥60 but ≤75 years
- Show signs of subjective cognitive decline (assessed by scoring ≥1 on the Subjective Cognitive Decline 9-item (SCD-9) questionnaire and endorsing “Yes, this worries me” on the subjective cognitive decline with worry item)
- Have the ability to make and receive phone calls
- Have the ability to send and receive text messages
- Have access to a desktop computer, video-teleconferencing and reliable internet connection
- Motivated to use a daily coaching program

Exclusion criteria

- Have a significant history of mental illness, substance abuse, learning disability, or neurologic conditions
- Have a history of dementia
- Have ophthalmologic or visual problems that prevent viewing a computer screen at a normal distance (eg, legal blindness, detached retinas, occlusive cataracts)
- Currently participating in a formal cognitive-training coaching program
- Currently pregnant

### Virtual Cognitive Health Program

The VC Health program comprises two phases: a 6-month active phase of lifestyle change and a 6-month maintenance phase of habit reinforcement. The individually tailored intervention encourages a healthy diet, physical exercise, cognitive training, and social engagement, all of which are supported by a coach who is reachable via telephone, email, and text messaging. To supplement all coaching interactions, we provided participants with Web-based psychoeducational material to help guide and pace individual learning.

#### Health Coaching

We assigned each participant a health coach for the duration of the intervention. All coaches were certified as personal trainers through nationally accredited programs, where the basic level of certification requires mastery of exercise physiology safety and nutritional health practices. To further ensure safety, a VC Health program nurse was available to assist coaches with the more complex behavioral health needs of participants.

After participants completed baseline testing, they were assigned a coach and completed an initial 1-hour phone call to discuss more detailed information about their current exercise and dietary habits. During the first 6 months of the intervention, participants had the option of scheduling weekly phone calls with their coach to discuss questions, difficulties, or adjustments to lifestyle behaviors. After the first 6 months, we gave participants the option to maintain the weekly cadence of coaching calls or to reduce the cadence to biweekly or monthly calls. We offered the option to adjust the call frequency to accommodate the varying levels of self-efficacy that the participants developed throughout the intervention.

#### Physical Exercise

All participants received psychoeducation regarding the benefits of physical exercise, such as aerobic and bodyweight training, on cognitive health. As part of the intervention, we also provided participants with Fitbit Flex 2 wearable devices (Fitbit, Inc, San Francisco, CA, USA) to help track and monitor activity levels. Participants were encouraged to log all exercise data in electronic trackers on the VC Health program platform. In an effort to prevent overwhelming participants with too much educational content at once, we prioritized exercise for the first month, prior to coaching for diet or cognitive training.

Health coaches assisted participants with creating individually tailored physical activity programs that incorporated aerobic exercise and progressive muscle strength training. Individual aerobic exercise plans prioritized activities preferred by each participant, such as swimming, biking, and walking. The exercise training program is a modified version of the FINGER study [15] physical activity intervention, including bodyweight strength training and aerobic exercise. The bodyweight strength training program included exercises for all primary muscle groups.

Coaches assessed each participant’s level of fitness at the beginning of the program and used the information to individually tailor exercise recommendations. Based on coach evaluations of self-reported exercise levels at baseline, participants were placed into low, moderate, and high categories. Low exercisers were those who completed aerobic exercise fewer than 3 times per week (minimum of 30 minutes per session) or no body resistance exercise (minimum 30 minutes). Moderate exercisers were those who completed aerobic exercise 3 times per week (minimum 30 minutes per session) or body resistance exercise fewer than 2 times per week (minimum 30 minutes per session). High exercisers were those who completed aerobic exercise 4 or more times per week (minimum 45 minutes per session) and body resistance exercise 2 or more times per week (minimum 45 minutes per session). Participants were encouraged to set a goal of reaching the next exercise threshold throughout the program or sustaining current levels if they were categorized as high exercisers at baseline.

#### Diet and Nutrition

**Figure 1:**
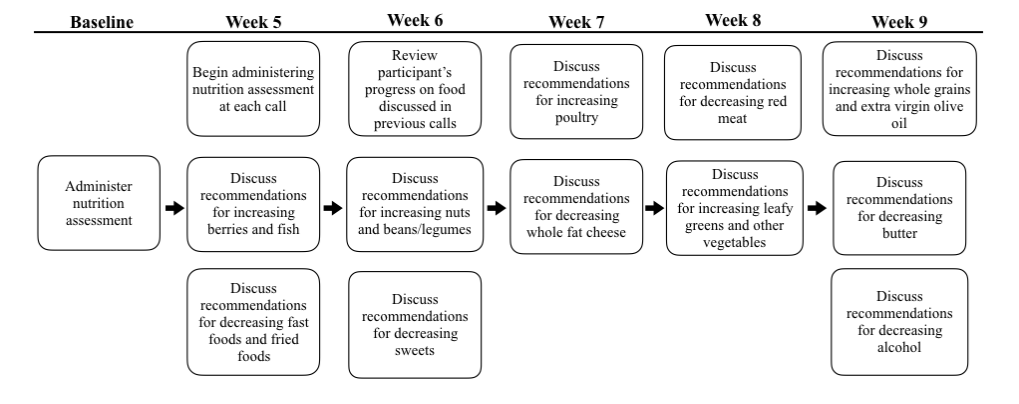
Flow for assessing and improving participant dietary habits.

All participants received psychoeducation on the benefits of the Mediterranean-Dietary Approach to Systolic Hypertension (DASH) Intervention for Neurodegenerative Delay (MIND) diet for cognitive health. The MIND diet emphasizes the consumption of foods that have been shown to have positive effects on cognitive health [27]. Combining pieces of the Mediterranean and DASH diets, the MIND diet recommends regular consumption of berries (≥5 servings per week), fish (≥1 servings per week), nuts (≥5 servings per week), beans or legumes (≥4 servings per week), poultry (≥2 servings per week), green leafy vegetables (≥1 servings per day), other vegetables (≥1 servings per day), grains (≥3 servings per day), and extra virgin olive oil (≥3 servings per day) [28]. The MIND diet recommends limited consumption of fried and fast foods (≤1 servings per week), sweets (≤5 servings per week), whole fat cheese (≤1 servings per week), red meat (≤4 servings per week), butter (≤1 servings per day), and alcohol (≤2 servings per day) [28]. During the initial coaching call, participants were assessed on their current fidelity to the MIND diet and were categorized as either high adherers (meets >7 MIND recommendations) or low adherers (meets ≤7 MIND recommendations). Coaches used these categories as a baseline for guiding participants through increasing MIND diet adherence (Figure 1), helping each participant create an individually tailored diet plan.

#### Cognitive Training

We provided participants with a curriculum on the benefits of cognitive training for cognitive health, including a library of curated content on the topic. VC Health program coaches helped participants create an individually tailored cognitive training program. The training program was provided by a Web-based service (MindAgilis, London, England) and included several tasks adapted from protocols previously shown to be effective in shorter-term RCTs, focusing on processing speed, executive function, working memory, episodic memory, and mental speed [29,30].

#### Social Engagement

All participants received access to an internal, private social network where they could engage in communal support and directed life review. Participants in the study were given the opportunity to connect with one another and were also able to invite one family member and one friend to join the social network. Participants were asked to respond to a variety of discussion prompts, including structured life review questions based on an evidence-based protocol [31,32], and participate in discussions about other study participants’ life review reflections. The social network was moderated by a clinical psychologist.

### Neurotrack Imprint Eye-Tracking Test

As part of the intervention, we asked participants to complete the Neurotrack Imprint eye-tracking test as an additional measure of cognition [33]. The test consists of a 5-minute visual paired-comparison task developed by Neurotrack Technologies, Inc. (Redwood City, CA, USA). Visual paired-comparison tasks quantify how the test participant splits attention between familiar and novel visual stimuli, with a familiarization phase preceding a testing phase. During the familiarization phase, participants are presented with pairs of identical visual stimuli for a fixed period of time (5 seconds). During the test phase, which follows a delay of either 2 seconds or 2 minutes to assess immediate and delayed recognition memory, participants are presented with additional pairs of visual stimuli, including one from the familiarization phase and one novel stimulus. The ratio of time participants spend gazing at the novel stimulus relative to the total viewing time produces a novelty preference score, with higher scores representing better declarative memory function [34]. Test-retest reliability (*r* range .88-.92) and interrater scoring agreement (κ range .81-.88) for the Imprint test have both been documented as high based on previous literature [33].

### Outcome Measurements

With cognition as the primary focus of this investigation, the RBANS was remotely administered [35] to all participants by qualified clinicians with experience in digital delivery at baseline (week 0), week 12, week 24, and week 52. The RBANS has demonstrated strong efficacy as a dementia assessment tool in community-dwelling normal individuals [36,37] and can also detect cognitive impairment associated with Alzheimer disease [38]. The primary outcome in this study was change in RBANS total score from study baseline to week 24 and week 52.

We assessed secondary risk factors for Alzheimer disease (depression and anxiety) through the PHQ-9 and GAD-7 scale at baseline, week 12, week 24, and week 52. We chose these items because depression [39-41] and anxiety [42,43] are predictive of future cognitive decline, with symptoms of both tending to manifest before direct evidence of cognitive decline is present. The PHQ-9 is a self-administered version of the Primary Care Evaluation of Mental Disorders (PRIME-MD) diagnostic instrument for common mental disorders [44]. The PHQ-9 comprises the depression module, which scores each of the 9 *Diagnostic and Statistical Manual of Mental Disorders* (Fourth Edition) criteria for depression from 0 (not at all) to 3 (nearly every day) and has been validated for use in primary care [45]. The GAD-7 is a self-report anxiety questionnaire designed to assess anxiety status during the previous 2 weeks. The items in the questionnaire assess the degree to which an individual has been bothered by nervous, anxious, or on-edge feelings; lack of ability to stop or control worrying; worrying too frequently about various things; inability to relax or sit still; ease of becoming annoyed or irritable; and feeling afraid [46]. The secondary outcome of this study was change in PHQ-9 and GAD-7 scores from study baseline to week 24 and week 52.

In addition to depression and anxiety symptoms, we asked participants about their sleep (hours per night) and exercise (days per week) habits during the previous 3 months. We collected these data at the same time points as the PHQ-9 and GAD-7 results. At 24 and at 52 weeks, we asked all participants to provide subjective data on their perceived improvements in cognitive ability, physical activity levels, eating habits, sleep patterns, and stress levels. Exploratory analyses will examine changes in self-reported behaviors and how engagement with the VC Health program is associated with change in RBANS performance.

Lastly, we will assess a number of feasibility outcomes to inform future study designs and program iterations. For one, we will analyze the ease of recruitment and the study retention rate. These results will provide rough estimates of what we can expect in future trials on the VC Health intervention. We will also analyze qualitative data about the participant experience in the intervention that we collected through online surveys at 12, 24, and 52 weeks. The feedback from participants will shape future program features and help to optimize the participant experience.

### Sample Size and Statistical Analysis

Given the preliminary nature of this study, we did not power the study to detect any specific difference in RBANS score, and we determined the sample size based on intervention capacity. Ultimately, 85 participants enrolled into the study.

Analyses will be conducted on deidentified aggregate data from the intention-to-treat population. The primary analysis will explore mean and median change in RBANS score from study baseline to the 24-week and 52-week primary end points. For both the RBANS and online assessments, we included the 12-week time point to allow for an interim nonprimary analysis early on in the study. We will also examine the mean and median changes stratified by key participant characteristics, such as sex, age, and education. Secondary analyses will examine the mean and median change in PHQ-9 and GAD-7 scores from study baseline to 24 and 52 weeks. Potential supplemental analyses will examine various measures of user engagement, the relationship between engagement and changes in RBANS scores and secondary outcome measures, and changes in various lifestyle behaviors, such as sleep and exercise habits.

## Results

**Figure 2:**
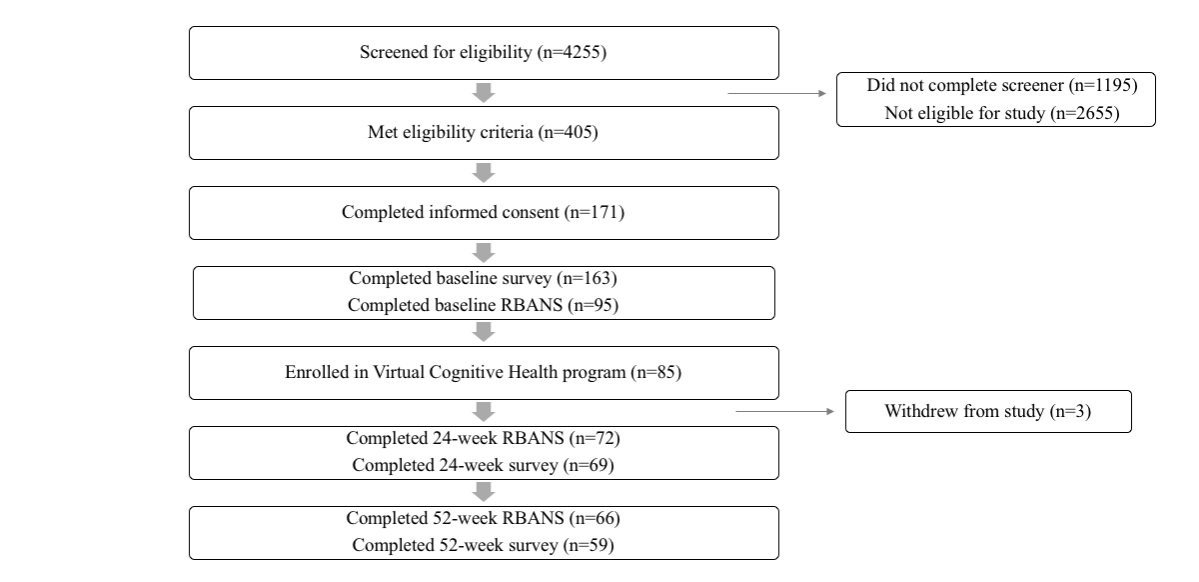
Enrollment cascade and study timeline. RBANS: Repeatable Battery for the Assessment of Neuropsychological Status.

Study recruitment, screening, and enrollment took place between November 2016 and March 2017. A total of 4255 participants were identified as potentially eligible from recruitment strategies and initiated the screening process. Of these, we determined 2655 to be ineligible based on factors including baseline cognitive function, history of mental illness, vision issues, and current pregnancy. Out of the final 405 individuals deemed eligible, 85 individuals provided informed consent, completed baseline surveys, finished all baseline assessments, were assigned a health coach, and started the VC Health program. Of these, 3 individuals withdrew from the study due to personal reasons, leaving 82 individuals eligible for data analysis (Figure 2).

**Figure 3:**
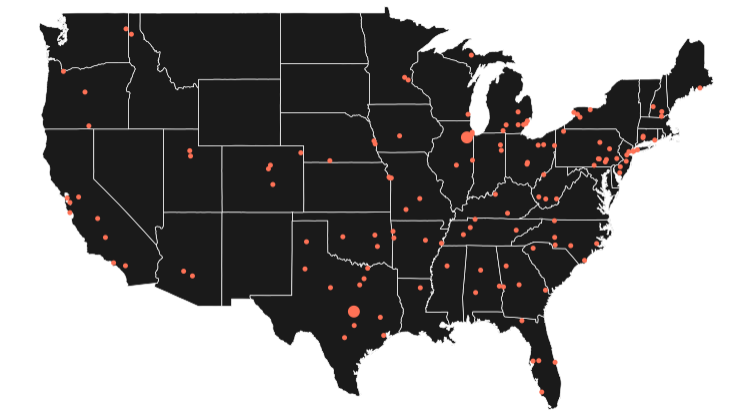
Geographic distribution of study participants. Each dot on the map corresponds to a study participant’s zip code. Larger dots represent multiple individuals from that zip code.

Table 1 displays the baseline demographic characteristics of study participants. Overall, 61 of the 82 participants (74%) were female, and the mean age was 64 years (range 60-74.9). Of the 82 participants, 72 (88%) identified as white, 5 (6%) as African American, 3 (4%) as Hispanic, 1 (1%) as Asian, and 1 (1%) as other. Of the 82 participants, 55 (67%) had a college degree or higher. Average baseline body mass index was 30.7 (SD 7.0) kg/m^2^, with an average weight of 87.5 (SD 20.8) kg. Enrolled participants represented a geographically diverse population (Figure 3).

At baseline, the average total RBANS score was 95.9 (SD 11.1), which is within normal age-adjusted ranges. The average SCD-9 score was 6.0 (SD 2.0), indicating minor subjective cognitive decline and, as previously mentioned, all participants endorsed worry about cognitive decline. The average GAD-7 score was 6.2 (SD 4.5) and the average PHQ-9 score was 8.5 (SD 4.9), respectively indicating mild levels of anxiety and depression at baseline (Table 2).

**Table 1:** Baseline demographics of enrolled participants (N=82).

Characteristics		Value
Age (years), mean (SD)		64 (4)
**Sex, n (%)**		
	Male	20 (24)
	Female	61 (74)
	Other	1 (1)
**Education, n (%)**		
	High school graduate or GED^a^	3 (4)
	Trade, technical, or vocational training	2 (2)
	Some college, no degree	22 (27)
	College graduate, associate’s or bachelor’s degree	29 (35)
	Graduate degree	19 (23)
	Doctorate	7 (9)
**Race/ethnicity, n (%)**		
	African American	5 (6)
	Asian	1 (1)
	White	72 (88)
	Hispanic	3 (4)
	Other	1 (1)
**BMI^b^ (kg/m^2^), n (%)**		
	<18.5 (underweight)	0 (0)
	18.5-24.9 (healthy weight)	16 (20)
	25.0-29.9 (overweight)	24 (29)
	30-34.9 (obese)	25 (30)
	≥35 (very obese)	15 (18)
**Average nightly sleep duration (hours), n (%)**		
	<3	1 (1)
	4-5	4 (5)
	5-6	18 (22)
	6-7	35 (43)
	7-8	16 (20)
	8-9	6 (7)
	9-10	0 (0)
	>10	6 (2)

^a^GED: General Education Development.

^b^BMI: body mass index; 2 participants were excluded due to BMI >60 kg/m^2^.

**Table 2:** Baseline assessment scores of enrolled participants (N=82).

Assessment instruments		Mean (SD)
**Repeatable Battery for the Assessment of Neuropsychological Status**		
	Immediate Memory	99.4 (12.5)
	Delayed Memory	96.6 (12.5)
	Visuospatial/Constructional	90.4 (14.3)
	Language	97.2 (10.2)
	Attention/Processing Speed	102.8 (14.8)
	Total	95.9 (11.1)
Subjective Cognitive Decline 9-item questionnaire		6.0 (2.0)
Patient Health Questionnaire 9-item		8.5 (4.9)
Generalized Anxiety Disorder 7-item scale		6.2 (4.5)

## Discussion

### Principal Findings

The VC Health study will investigate the feasibility and effectiveness of a remotely delivered multidomain lifestyle intervention for the prevention or delay of cognitive impairment in older adults with subjective cognitive decline. As the digital delivery of these interventions is a new area of study, validation of this format is needed to ensure similar effectiveness to in-person options. The results of this pilot study will provide preliminary insights into the translation of a traditionally in-person multidomain lifestyle intervention to a fully digital format and will help shape future program iterations. While face-to-face interventions like the one used in the FINGER study have shown promise for decreasing risk of cognitive decline [12], participation in these types of programs is constrained by geographical and logistical complications, such as scheduling conflicts and access to transportation. The only requirement for participation in a digital lifestyle intervention like VC Health is access to an internet-connected device, such as a mobile phone, tablet, or computer. For those who do not own such devices, the program may still be accessible by using technology that is available at local libraries and community centers, which further expands the potential reach of the program. The successful translation and adoption of digitally delivered lifestyle interventions has the potential to reduce the incidence of Alzheimer disease and medical spending on the disease as the search for effective pharmaceutical agents continues.

Digital lifestyle interventions have been developed for a variety of health-related conditions. These multipronged interventions have shown efficacy for diabetes prevention [47,48], diabetes management [49-52], cardiovascular risk reduction [53-55], pain management [56], and smoking cessation [57]. Many of the core components of lifestyle interventions are similar, independent of the health condition they address. These components generally include psychoeducational material, social support from a peer group, access to a health coach, and tracking tools to facilitate the adoption of new health behaviors. The VC Health program contains all of these features, with adjustments tailored to individuals at risk for cognitive decline. Some of the unique aspects of the VC Health program are the addition of cognitive training exercises [58-60], specific dietary recommendations for following the MIND diet [27,28,61,62], and validated physical activity recommendations specific for enhancing cognitive function [63-65]. Based on the successful translation of other lifestyle programs into a digital format, the VC Health intervention should lend itself well to online delivery.

While the adoption of Web-based lifestyle change programs has increased over the years, one of the main barriers to the widespread adoption of remote cognitive tests is the concern about data integrity. The remote delivery of cognitive tests makes it difficult to accurately monitor patients for effort, focus, and test understanding, which is normally completed by an in-person test administrator. However, previous literature has demonstrated the efficacy and effectiveness of remote RBANS delivery via videoconference [35], supporting its use in our investigation. The Imprint test, which is embedded in the VC Health program, uses webcam-based eye-tracking data, with recorded video from the test providing analysts with “eyes on the patient.” This allows the analysts to assess various elements of data quality and fidelity to test-taking procedures [33]. Remote administration of the RBANS and Imprint tests allows for the feasible and scalable collection of data at multiple time points and for the longitudinal monitoring of cognitive health. This investigation is, to our knowledge, the first of its kind in the cognitive health space, and is structured to demonstrate that a digital intervention can be delivered from baseline to completion while measuring primary, secondary, and exploratory outcomes with entirely remote mechanisms.

### Strengths and Limitations

The design of the VC Health study has both strengths and limitations. One strength is the digital administration of the intervention and collection of data in a real-world setting, which provides more ecologically valid results than studies completed in traditional research settings [22]. This is important, as it enhances the generalizability of the results and provides more powerful estimates of how the intervention is likely to perform in broader populations and settings. Another strength is the 52-week study duration, as this allows the results to reflect the long-term impact of the intervention. Examining the long-term outcomes of lifestyle change programs is essential to demonstrate the true impact of participation after the program tapers off or ends.

Limitations of this study include the small sample size and lack of a control group, which were both a result of the pilot nature of this investigation. However, it should be noted that intervention studies evaluating digital lifestyle programs commonly employ single-arm designs, and this design is well accepted in the field for early studies [47,48,52,66,67]. Lastly, the study sample was primarily white and well educated, so the results may not be generalizable to other populations. Future studies require the exploration of similar interventions in a more diverse group of individuals.

### Conclusion

This single-arm pre-post pilot study will provide initial evidence of the feasibility and effectiveness of a remotely delivered multidomain intervention to prevent or delay cognitive decline in older adults at risk for dementia. We will collect qualitative information on specific intervention components and use it to inform the ongoing design iterations of the Web-based intervention, as well as the design of larger studies investigating the effect of the intervention. The 24- and 52-week longitudinal follow-up periods used in this fully remote study will be the first to evaluate the effectiveness of a digital intervention to prevent or delay cognitive decline in older adults. In addition to composite cognitive performance, assessment of symptoms of anxiety and depression will allow us to explore the effects of the intervention on other aspects of mental health. We expect the results of this trial to provide crucial insights into the promise of remotely delivered cognitive health interventions, which could have a substantial impact on dementia incidence over the coming decades.

## Abbreviations

DASH: Dietary Approach to Systolic Hypertension
FINGER: Finnish Geriatric Intervention Study to Prevent Cognitive Impairment and Disability
GAD-7: Generalized Anxiety Disorder 7-item
MIND: Mediterranean-DASH Intervention for Neurodegenerative Delay
MIND-CHINA: Multimodal Intervention to Delay Dementia and Disability in Rural China
PHQ-9: Patient Health Questionnaire 9-item
PRIME-MD: Primary Care Evaluation of Mental Disorders
RBANS: Repeatable Battery for the Assessment of Neuropsychological Status
RCT: randomized controlled trial
SCD-9: Subjective Cognitive Decline 9-item
SINGER: Singapore Intervention Study to Prevent Cognitive Impairment and Disability
US POINTER: United States Study to Protect Brain Health Through Lifestyle Intervention to Reduce Risk
VC Health: Virtual Cognitive Health
